# Barriers and Facilitators Towards Deceased Organ Donation: A Qualitative Study Among Three Major Religious Groups in Chandigarh, and Chennai, India

**DOI:** 10.1007/s10943-024-02148-8

**Published:** 2024-10-06

**Authors:** Britzer Paul Vincent, Vibhusha Sood, Srinivasan Thanigachalam, Erica Cook, Gurch Randhawa

**Affiliations:** 1 Institute for Health Research, Faculty of Health and Social Sciences, University of Bedfordshire, Putteridge Bury Campus, Hitchin Road, Luton, LU2 8LE UK; 2 OHUM Healthcare Solutions Private Ltd, Pune, 411014 India; 3 Community and Family Medicine, All India Institute of Medical Sciences (AIIMS), Madurai, Tamil Nadu 625006 India; 4 Department of Psychology, University of Bedfordshire, Vicarage Street, Luton, LU1 3JU UK

**Keywords:** Religion, Religious leaders, Deceased organ donation, India, Transplant

## Abstract

This study aimed to examine the barriers and facilitators of the public toward deceased organ donation in Chandigarh, and Chennai, India, from three major religious groups, Hinduism, Islamism, and Christianity. Twenty-five focus groups were conducted (*n* = 87) stratified by study region, religion, sex, and age. Data were analysed using framework analysis. The results revealed that individuals were primarily willing to donate their organs. However, their religious views regarding death, after-life beliefs, funeral ritual practices, and lack of knowledge regarding their religion’s position toward deceased organ donation created tension and ambiguity in the decision-making. However, younger age groups (18–30 years) appeared more open and positive toward deceased organ donation. The conclusion demands a clear need for religious leaders and stakeholders to address their religion’s stance, which creates tension and ambiguity in any uncertainties surrounding cultural and religious-based views among the Indian population.

**Supplementary Information:**

The online version contains supplementary material available at 10.1007/s10943-024-02148-8.

## Introduction

Organ transplant practices, from live and deceased organ donation, have transitioned from an experimental practice to an established clinical practice globally and in India (Kute et al., 2020; Sulania et al., 2016). However, since the first organ transplant in India, transplantation practice has predominantly relied upon living donation compared to deceased organ donation (Abraham et al., 2013). Evidence from Indian National Organ and Tissue Transplant Organisation (NOTTO) reported that only 7% of the transplantation in India were from deceased organ donation (Organ Report, 2024).

A recently published integrative systematic review that explored the barriers toward deceased organ donation from the Indian public’s standpoint revealed that there are a range of complex barriers and facilitators which influences their views toward deceased organ donation, one factor being religion (Vincent et al., 2022). India is a multi-religious population, with the majority from Hindu background (79.8%), followed by Muslims (14.2%) and Christians (2.3%) (Census of India, 2011). While quantitative studies undertaken in India ‘statistically’ and ‘objectively’ measured the influence of religion on deceased organ donation, studies identified in a recently published systematic review have not demonstrated the public’s subjective views on how it influenced their attitudes toward deceased organ donation (Dasgupta et al., 2014; Vijayalakshmi et al., 2016).

While deceased organ donation has various lens to look at, such as ethical, social, political, and philosophical, the role of religion also needs to be examined among the public, such as how the public’s views or beliefs toward their respective religion influence their deceased organ donation behaviour and decision. Not all religions view deceased organ donation similarly (Doerry et al., 2022). While Christianity and Islamism are identified to have the same root in Abrahamic origin, their views are incredibly varied (Tarabeih et al., 2022). This is particularly interesting with deceased organ donation, as it deals with the definition of ‘death’ from a religious standpoint. Especially, deceased organ donation is viewed more critically among Muslims and Hindus than among Christians (Alhawari et al., 2020; Pradeep et al., 2019). Though the three religions spiritually view death as the dissociation of soul or spirit from the physical body (Miller et al., 2014), the uncertainty of when the spirit dissociates from the body has been a critical point in declaring death for some Muslim leaders, which leaves a minor group of scholars, undecided toward deceased organ donation (Miller et al., 2014).

In the Indian population, decisions on deceased organ donation are not solely under the control of an individual’s views alone, but based upon collective views, especially influenced by their religious views (Vincent et al., 2022). Hence, there is a greater need to identify from the Indian public’s perspective how their religious views influence their decision toward deceased organ donation. Therefore, this study develops with that aim to identify how the views of the public in India from three major religious groups such as Hinduism, Islamism, and Christianity, influence their perspectives toward deceased organ donation.

## Material and Method

This manuscript follows the consolidated criteria for reporting qualitative (COREQ) research: A 32-item checklist for interviews and focus groups (Tong et al., 2007) (Supplementary file 1).

### Study Epistemology

Among the Indian population, decision toward deceased organ donation is not individualistic but majorly collectivist in their approach (Vincent et al., 2022), identified to be similar across various Asian countries (Li et al., 2019; Siraj, 2022). Therefore, the epistemological lens to knowing the truth toward the phenomenon in this study adopted a social constructivism approach based on the worldview that knowledge is socially situated, whereby individuals seek to understand their world and develop meanings through interaction with others, religion in this instance (Creswell & Poth, 2016).

### Study Setting

Data on deceased organ donor registration and consent rates in India suggest that the southern region of India has notably performed better when compared to the northern region (Kute et al., 2021). Two studies which measured the consent rates in India for deceased organ donation showed that Chandigarh, a city from northern India, had a consent rate of 8.2% compared with Chennai, a city from southern India, with a consent rate of 64.8% (Kumar et al., 2014; Niranjan et al., 2014). Therefore, based on the evidence that the registration and consent rates are higher in the southern region compared to the northern region, the study sites adopted for the present were Chandigarh and Chennai, India. Chandigarh and Chennai are capital region of northern states (Punjab and Haryana) and a southern state (Tamil Nadu) in India, respectively (Census of India, 2011). Tamil Nadu was the first state to initiate the cadaver transplant program in 2009 following the Spanish model, making them a pioneer in the field of deceased organ donation practices in India (Abraham et al., 2013). Following this, several other states in India followed their journey. While the Human Development Index (HDI) of both these study sites are similar (high HDI states) (Ministry of Statistics and Programme Implementation, 2019), the performance of deceased organ donation is highly varied. According to Patton (2014), this study site sampling strategy approach is called comparative purposive sampling. This sampling strategy argues for understanding the phenomenon of interest by comparing different study sites or clusters based on a criterion (i.e., deceased organ donation performance criterion in this study).

However, the study group settings were not just stratified based on the study sites but also based on religion (Muslim, Christian, Hindu), sex (male and female), and age (18–30 years and 30 years above). Stratification of the study group settings were undertaken based on a recent systematic review which informed that the barriers and facilitators toward deceased organ donation among Indians living globally are influenced by these factors (Vincent et al., 2022). This approach was also adopted as it enables and creates a secure and comfortable group for the participants to freely express their views (Kitzinger, 1994) and has been a common approach used in organ donation research (Dupper et al., 2015; Gauher et al., 2013; Joshi, 2011; Randhawa, 1998). Since the study was conducted via teleconferencing (mobile phone conference calls), the participants in the FGD groups could remain in their own homes while participating, and they were asked to avoid the presence of any other family members.

### Study Participants

Individuals aged eighteen years and older were included as this is the age of eligibility to register and consent for deceased organ donation in India (The Transplantation of Human Organs and Tissue Act, 1994). Only individuals who were residents of Chennai, and Chandigarh, India, were included in the study. There was no exclusion based on the socio-economic status of the participants. However, individuals with an occupational or educational background in healthcare were excluded, alongside individuals with any personal or family history of organ donation or transplantation, as they have the potential for deviated views from the public (Vincent et al., 2022). India is a multi-religious group, with Hinduism, Islamism, and Christianity representing 97% of the total Indian population (Census of India, 2011), and this study recruited only those who self-identified as being from one of these three religions (Table 1). Individuals who spoke Tamil or English from Chennai and Hindi, Punjabi, or English from Chandigarh were only eligible to be included, since those are their respective local and majorly used languages.

**Table 1:** Demography of participants (*N* = 87)

	*n*	(%)
*Study region*		
North–Chandigarh	44	(50.5)
South–Chennai	43	(49.5)
*Sex*		
Male	42	(48.3)
Female	45	(51.7)
*Age group (in years)*		
18–20	6	(6.9)
21–25	20	(23)
26–30	19	(21.8)
31–35	9	(10.3)
36–40	6	(6.9)
41–45	11	(12.6)
46–50	5	(5.8)
51–55	7	(8.1)
56–60	3	(3.5)
60 above	1	(1.1)
*Religion*		
Hindu	28	(32.2)
Muslim	29	(33.3)
Christian	30	(34.5)

### Participant Recruitment

In 2019, the first author of the present study who was a PhD student then, visited several organisations in India that worked with the community on various public issues. In addition to the collaboration with MOHAN (Multi-Organ Harvesting Aid Network) Foundation, India, network with several other organisations in India working for gender equality, farmers’ well-being, public health issues, educational institutes, and religious groups aided in participant recruitment. All these participants were never a part of any organ donation-related campaign, thereby reducing any biased views due to their pre-engagement with organ donation. Participants were looked for based on the characteristic of the focus group discussions (FGDs) stratified based on region, religion, sex, and age. Once potential participants were identified, the participant information sheet was vocally read out to the participants over telephone calls, and consent was audio-recorded, a similar ethical approach adopted in other qualitative studies undertaken during the COVID-19 pandemic (Kumari et al., 2021). Later, the date and time for the focus group discussion were fixed based on the availability of their respective group members.

### Data Collection

The FGDs are particularly helpful in studies undertaken to understand public attitudes toward organ donation, as participants often lack direct exposure to the phenomenon (Albright et al., 2005; Ralph et al., 2016; Wong, 2010). This method was also adopted as it encourages interaction with the co-participants, which resembles a similar interaction in the society where the phenomenon is constructed through these social interactions, thereby aligning with the epistemology of the study (Wilkinson, 1998).

A total of twenty-five FGDs were conducted, with eighty-seven participants (Table 2) during the COVID-19 pandemic period. The FGDs lasted between sixty and ninety minutes. Given the pandemic and restrictions on social distancing during data collection, the FGDs were conducted using a teleconferencing platform (mobile phone conference calls). This is a widely used method when the researcher and the participants are displaced by space and time (Cooper et al., 2003), as was the case for this study (Omer et al., 2022).

**Table 2:** Demography of the FGDs

Region	Religion	Age	Group	Number of participants per group	Language used	Facilitator
Chandigarh (Northern study site)	Hindu	18–30 years	Male group	3	Hindi	V.S
			Female group	4	Hindi	V.S
		30 years above	Male group	4	Hindi	V.S
			Female group	4	Hindi	V.S
	Muslim	18–30 years	Male group	4	Hindi	V.S
			Female group	3	Hindi	V.S
		30 years above	Male group	3	Hindi	V.S
			Female group	4	Hindi	V.S
	Christian	18–30 years	Male group	4	Hindi	V.S
			Female group	4	Hindi	V.S
		30 years above	Male group	3	Hindi	V.S
			Female group	4	Hindi	V.S
Chennai (Southern study site)	Hindu	18–30 years	Male group	4	Tamil	B.P.V
			Female group	3	Tamil	B.P.V
		30 years above	Male group	3	Tamil	B.P.V
			Female group	3	Tamil	B.P.V
	Muslim	18–30 years	Male group	3	Tamil	B.P.V
			Female group	4	Tamil	B.P.V
		30 years above	Male group	4	Tamil	B.P.V
			Female group	4	Tamil	B.P.V
	Christian	18–30 years	Male group	4	Tamil	B.P.V
			Female group	2	Tamil	B.P.V
			Female group	3	Tamil	B.P.V
		30 years above	Male group	3	Tamil	B.P.V
			Female group	3	Tamil	B.P.V

B.P.V (principal investigator) and V.S (research assistant) represent the authors who facilitated the respective FGDs

Due to technical and feasibility issues and to enable participants to talk more openly and freely using teleconferencing platforms (Kruger, 2014), good practice guidelines suggest the size of the groups should be smaller than traditional focus groups, between three and five participants per group (Appleton et al., 2000; Cooper et al., 2003). Based on this evidence, the size of FGDs in this study varied between three and four participants per group (Table 2). Therefore, smaller in size per group compared to traditional FGDs, while appropriate for the platform used (Appleton et al., 2000; Cooper et al., 2003).

The researcher, B.P.V has a master’s in public health and undertook this study as part of his PhD research. The FGDs in the south of India were facilitated by B.P.V, who is from the local region and is fluent in Tamil (i.e., the local language of Chennai). The FGDs in the north of India were facilitated by V.S, a bilingual female research assistant (RA) fluent in Punjabi and Hindi (i.e., the local language of Chandigarh), with a master’s in public health and experience in conducting qualitative research. Both B.P.V and V.S are from their respective fieldwork community. This enabled good rapport with the participants as they also spoke the local language, thereby addressing the authenticity of the study. However, the facilitators had no prior connection with any of the participants who took part.

The questions used in the study were based on several similar organ donation studies published prior (Dupper et al., 2015; Gauher et al., 2013; Joshi, 2011; Randhawa, 1998) and developed to align with the gaps identified in a recent organ donation systematic review among Indians living globally (Vincent et al., 2022) (Table 3). Before data collection, the FGDs were pilot tested by both the facilitators (B.P.V and V.S). The background knowledge, perspectives toward organ donation, or religious viewpoints of the facilitators (B.P.V and V.S) were controlled by using neutrally phrased questions for the discussions (Table 3). Therefore, confirmability was attained by using neutral questions and prompts to guide the study, and all the findings were based descriptively on the participants’ quotes to confirm the findings (Guba, 1994; Lincoln & Guba, 1985).

**Table 3:** Focus group discussion guide

1. What are your views toward deceased organ donation?
Probe:
i. Would you be willing or not willing to register or donate organs when dead? Why?
2. Do you think that religion may influence or affect your decision?
Probes:
i. If yes, could you elaborate how and why it influences, please?
ii. If no, could you elaborate how and why it does not influence, please?
iii. If no, then why do you think people say cultural or religious issue as a challenge to donate the organs of their loved ones?
3. Do you think that you will give importance to your religion in regard to deceased organ donation?
Probes:
i. If yes, why do you give importance?
ii. If no, why do you give no or less importance?
4. What do you think that your religion may have to say when you decide to register in the organ donor list or give consent to donate the organs of your loved ones after their death?
Probe:
i. Can you explain me more to have a better understanding, please?
5. Do you think that body or any funeral practices may influence your decision toward deceased organ donation?
Probes:
i. If yes, can you explain me more to have a better understanding, please?
ii. If no, can you explain me more to have a better understanding, please?

### Data Analysis

All the FGDs were audio-recorded with the participant’s permission, and translated and transcribed in English by the facilitators (B.P.V for south Indian FGDs and V.S for north Indian FGDs). In alignment with the aim, purpose, and sampling technique, the present study adopted framework analysis (FA) to analyse the data (Ritchie & Spencer, 2002). This was based on the purpose of allowing and enabling case comparison and association between- and within- groups (Ritchie and Spencer, 2002). It followed five steps, namely: (1) familiarisation, (2) identifying a thematic framework, (3) indexing, (4) charting, and (5) mapping and interpreting.

The data coding was undertaken by three coders using NVivo 11. Apart from the first author (B.P.V) and the RA (V.S), a second RA (S.T) was also recruited who was a bilingual male from south India with public health research background for transcribing a few transcripts from Chennai, and cross-coding the transcripts from both Chennai and Chandigarh. Employing three independent coders helped mitigate the implications of subjectivity while coding, more dependable findings by intercoder reliability, consistency in coding decisions, reflexivity, and confidence that the interpretation is beyond the individual researcher (Church et al., 2019). Such intercoder reliability in cross-cultural qualitative studies has demonstrated confidence in the varied language and cultural contexts (Joffe & Yardley, 2003; O’Connor & Joffe, 2020).

Identifying an appropriate study site, appropriate sampling frame, collection method, and data analysis method aligned with the aim of the research made the study highly authentic (Guba, 1994; Lincoln & Guba, 1985). Therefore, all these measures increased the rigour and trustworthiness of the study. Data sufficiency for the study was obtained at the level of religion, sex, and age of the study sample. Data sufficiency at this level demonstrated transferability of the evidence while the participants from almost similar groups, either based on religion, sex, or age, demonstrated repetition of information across (Guba, 1994; Lincoln & Guba, 1985).

## Findings

**Fig. 1:**
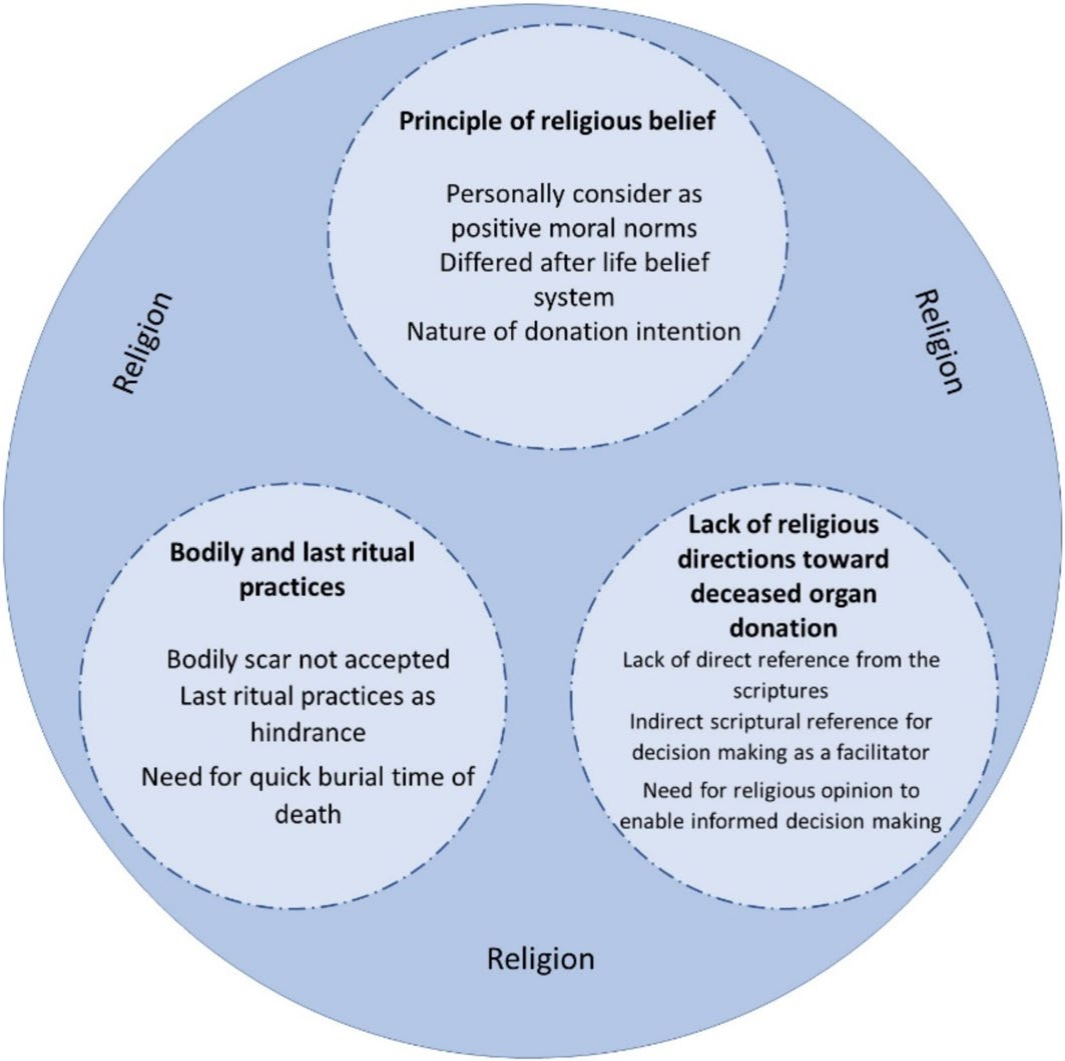
Themes and sub-themes on how religious perception constructs the attitudes toward deceased organ donation

The findings revealed that whilst individuals were primarily willing to donate their organs, there were three themes from a religious lens identified to influence their view toward deceased organ donation, namely: (1) principles of religious belief, (2) bodily and last ritual practices, and (3) lack of religious direction toward deceased organ donation (Fig. 1). These concepts created some tension and ambiguity among the participants in various ways.

### Theme 1: Principles of Religious Belief

*Description* This theme describes how the religious belief and principles held by the participants of the three religious groups influence their views toward deceased organ donation.

In all the FGDs, while not thinking about religion behind their mind, based on their sole personal view, irrespective of their age, sex, religion, and the study site, informed that deceased organ donation is a selfless act of saving another person’s life and personally considered it a positive moral norm. *“I feel organ donation is a noble act. I also feel that organ donation is the right thing to do. It is a noble act and an act of giving life to others. It is a better option.”*—Hindu female, 30 years above, from Chennai.

However, when further moved into the discussion, the pattern of interconnection between their personal views and their religious beliefs on death and after-life influenced their decisions, and conflicting views were identified only in the Hindu and Muslim FGDs. Consistent pattern of beliefs in death and after-life in favour of deceased organ donation was identified among the 18–30 years Hindu FGDs. *“First of all, I don’t think religion will matter for me. Some people [referring to older generation] think that you will be reincarnated without the donated body parts. Though, I think religion do influence people’s decision, but not for our generation.”*—Hindu female, 18–30 years, from Chandigarh. This belief was not consistent among the participants in the 30 years above groups. While some of the 30 years above participants held the same views of the 18–30 years group, most of the others differed. *“I [context: very few participants] don’t believe in such thoughts [counter arguing the other majority participant’s views within the groups] like we will not have intact organs after rebirth. I think Hinduism believes that soul is immortal, and body is mortal. Our body will not go with us after death.”*—Hindu female, 30 years above, from Chennai.

Predominantly, among all the 30 years above Muslim groups, there was a consistent conflicting pattern between the favourable *‘personal views’* and what they think the *‘religious leaders views’* would be, which influenced an unfavourable decision. *“I think according to Islam [context: assuming the views of their religious leaders] we can’t donate the organs after death because I think religious scholars don’t agree for organ donation, as we have to return the same body to Allah.”*—Muslim male, 30 years above, from Chandigarh. This was in clash with most of the Muslim participant’s personal views favouring organ donation as a noble act that saves other lives. *“My personal opinion is that if I die, I will donate my organs. If my body is of use for somebody else, then it is very great for me.”*—Muslim male, 18–30 years, from Chandigarh.

### Theme 2: Bodily and Last Ritual Practices

*Description* This theme describes how bodily integrity and the last ritual practices following the death of an individual influences their views toward deceased organ donation. However, participants viewed this from a viewpoint of practical or feasibility challenges if they engaged in deceased organ donation practices.

Bodily scar was predominantly demonstrated as a barrier among the Muslim FGDs. However, they often referred it as their religious leader’s view and not their own personal view. *“I think religious scholars don’t agree for organ donation as we have to return the same body to Allah.”*—Muslim male, 30 years above, from Chandigarh. This pattern of belief was contrary to all younger age group Muslim participants. *“I think there is misconception in the society that we should send the body to God like how he gave us. I don’t think so that there is any strict law or rule or norm on this. I think we can donate the organs after death.”*—Muslim male, 18–30 years, from Chandigarh.

Both Christian and Muslim participants perceived that the last rites, including the ceremonial washing of the deceased, would be compromised. *“I think we cannot do our last rituals. This is because they have sutures around their body and are packed well in a white cloth. In that case, we cannot do the formalities like giving them bath, changing their clothes to their wedding clothes and all those things. I have not seen any event directly. However, I think this will be one of the challenges in making our decision to donate organs of our loved deceased ones”*—Christian female, 30 years above, from Chennai.

However, the discussion among the Hindu participants showed a different viewpoint on how the last ritual practices could be challenged by deceased organ donation, majorly demonstrated only in Chandigarh, India. *“I come from a religion where we burn the body and put those ashes of the body in the Ganges [i.e., the name of a river in north India considered to be holy for last rituals]. I think… for that complete parting, all the elements need to come together for the soul to be relieved from the worldly affairs. Therefore, I think this will be a challenge to decide.”*—Hindu female, 18–30 years, from Chandigarh. This was identified only among the focus groups from the north of India.

There was a need for quick burial following death, identified only in all the Muslim FGDs, which challenged their thought of deceased organ donation. *“After death, burial has to take place as soon as possible. It can’t delay for any reason, other than if any family relative is left who had not seen the body. In our religion, we cannot wait for a long time to bury the body, maximum what we can wait is up to one day for burial.”*—Muslim male, 30 years above, from Chennai.

### Theme 3: Lack of Religious Directions Toward Deceased Organ Donation

*Description* This theme describes about the influence of the lack of religious opinion or viewpoint toward deceased organ donation and how decision is made in such a situation.

This theme was majorly identified only among the Muslim and Christian focus groups. However, among all the three religious’ groups, dependency on religious views was highly seen among the Muslim participants, both from Chandigarh and Chennai, specifically among the 30 years above age group FGDs that could be sensed from the contexts below.

Participants only from the thirty years above Muslim and Christian FGDs mentioned references to scriptures while considering decisions on deceased organ donation. *“I have read Quran shareef also. I don’t know what Quran shareef says about organ donation. I think Islam runs on the basis of Quran shareef, and as per Quran shareef, there are no thoughts and there is nothing written on organ donation. There is silence about that.”*—Muslim male, 30 years above, from Chandigarh. Such a lack of clarity made organ donation decision challenging for these participants, majorly among Muslims. In such a situation, they often delved in indirect scriptural comparisons. However, such indirect decision-making was majorly supported by the younger age group discussions. *“In Quran, there is no specific verse which says if you can or cannot donate your organs. However, there is a verse which says that saving a person life is equal to saving a mankind. I think, in that view, organ donation is supported.”*—Muslim male, 18–30 years, from Chennai***.***

In contrary, the older age groups were highly dependent on the religious leaders’ views in such a situation. *“Our family follows religion strictly. If we go against religion, then it is a problem for the family also. But if religion allows, then my family will agree whatever religion says. However, to decide, I am not sure what the religious leaders’ views are.”*—Muslim male, 30 years above, from Chennai. Hence, engagement and visibility of religious leaders’ standpoint were positively looked for by the participants in all the FGDs. *“If there are some clarities on organ donation… clearly said by the religious leaders, it will be of great support to decide… If this and other supportive principles can be told, it would help a lot.”*—Hindu female, 30 years above, 18–30 years, from Chandigarh.

## Discussion

**Fig. 2:**
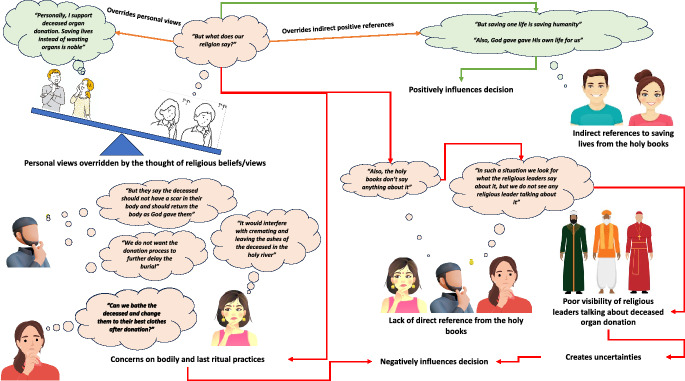
Summary of the influence of religious views

This study aimed to identify the barriers and facilitators toward deceased organ donation practices based on India's three major religious populations (Hinduism, Islamism, and Christianity). Every participant in the study perceived deceased organ donation as a noble act and was willing to be an organ donor. However, when further discussed, various religious perspectives were identified that negatively influenced and challenged their willingness to be deceased organ donors (Fig. 2).

Participants from all three-religion believed in the after-life in several ways, and this influenced their decision-making toward deceased organ donation. Christian participants expressed that they believed the soul (or spirit) and body to be two different entities, which helped them be more supportive of organ donor decision-making. On the other hand, among the Hindus, there were inconsistent beliefs, while some (majorly younger generation) believed body and soul to be two different entities and differed views in re-incarnation, other older Hindu participants rejected it. Similarly, there was a difference in views between the younger and older Muslim participants. Older generation believed that in-order to live with God and to respect His gift (i.e., the Body), they need to give it back as a whole. However, the younger generation were not willing to go against the will of their family members. This demonstrates the nuanced details of how the belief in the same concept—which is after-life among all three religions influences their organ donation decision in different ways. To improve deceased organ donation, such detailed considerations ought to be factored into interventions to be developed in addressing the concept of the afterlife (Ali et al., 2022; Randhawa & Neuberger, 2016) rather than knowledge alone.

While participants regarded deceased organ donation as a noble act from their own view and through their indirect interpretations from their holy books, certain ritual practices and beliefs confused their decision on how to proceed with deceased organ donation. For instance, the performance of washing the deceased among Muslims and Christians, quick burial among Muslims, and leaving the ashes in the holy river among Hindus influenced their decision. While religion was not blamed for being a direct barrier toward deceased organ donation, such ritual practices informed by their respective religion influenced their decision. However, despite this, several deceased organ donations and transplantation are already taking place among these population and sharing of information from the experience of the donor and recipient families or stakeholders from all these religious groups has the potential to enable the public to make a clear informed decision for themselves (Randhawa & Neuberger, 2016).

While individuals from all religions knew that organ donation was a noble act, they were not confident enough to decide without their religious leaders’ opinion. For instance, among all the Muslim focus groups, participants were worried that if they decided to donate the organs of their deceased loved ones, they might be criticised by their peers, if organ donation had not been supported by their Imams. A review of seventy fatwas informed that though there is a necessity to respect the body, organ donation is permissible in Islam to give life to fellowmen (Van Des Branden & Broeckaert, 2011). Although imams, gurus, and popes have expressed positive views on deceased organ donation, a lack of public awareness regarding their positions has led to a disconnect between personal beliefs and religious teachings, which in turn negatively impacts decision-making on this issue (Carey et al., 2011; Randhawa et al., 2012; Chiramel et al., 2020). Hence, such information must be disseminated among religious communities to enable an informed decision on deceased organ donation in India (Kute et al., 2020 and 2021).

A study among university students in a Muslim major country estimated that the willingness toward organ donation was 2.56 (CI 1.75–4.52) times higher than those unaware of their Imam’s views (Aghaee et al., 2015). A recent study demonstrated that religious intervention in informing a community exponentially changes their views and willingness to consider deceased organ donation (Ali et al., 2022; Sayyed et al., 2021) rather than campaigns on knowledge alone. Another recently published study showed that knowing the religious standpoint to be supportive toward deceased organ donation led the participants to be more willing (Krupic et al., 2019; Taş et al., 2022). Therefore, there is a greater need among the public toward informing the religious views on organ donation with more significant details rather than focusing the awareness campaign only on the component of knowledge and the need for organ donation.

Additionally, employing behaviour-influencing constructs, such as those from the health belief model, has been shown to positively impact deceased organ donation among the South Asian population in the UK (Pradeep et al., 2023). Addressing constructs like perceived susceptibility, benefits, and barriers from a religious standpoint, and using cues to action such as media visibility and normalising the conversation, has the potential to improve deceased organ donation rates in this population.

While the above challenges were identified toward deceased organ donation in this study, all the younger age groups participants from three-religion seemed to be more open for discussion and look to make the decision not solely based on religious views but how they personally feel about it, like few UK study findings among the younger age groups (Gauher et al., 2013; Joshi, 2011; Pradeep et al., 2019). Therefore, while religious intervention is needed among both age groups, the younger generation could be more visible in organ donation campaigns, like the UK NHS community engagement project approach, to bring forth this conversation in India.

Despite the significant detailed findings in the present study, they should be noted considering various limitations. While the number of FGDs in total was twenty-five groups, the actual number of participants in each group was between three and four per group. However, smaller groups in teleconferencing aided in rich FGDs, avoided overcrowding of views and technical issues, and enabled equal opportunity for all participants. While the present study tried to have equal opportunities for participants from various stratified categories in the sampling strategy, this was challenging with specific age groups, especially sixty years and above. Also, though individuals from Hinduism, Islamism, and Christianity were included, several sub-denominations exist within the same religion, which could have a different view. Therefore, while this study forms the foundation for the three major religions in India, further studies based on their respective sub-denomination will aid with an even deeper understanding (Tarabeih et al., 2022). The present study was undertaken in two specific regions in India, which varied in consent rate and donation number; therefore, the findings cannot be generalised to the larger population of India. However, this study aimed not to generalise the findings but to get rich in-depth evidence achieved by data sufficiency to answer the aim for a qualitative approach study.

## Conclusion

The findings highlight several differences in the barriers and practical issues around Hinduism, Islamism, and Christianity from the public’s view. Though interventions to improve organ donation among the public have primarily focused on increasing knowledge, the present study showed that the messengers who disseminate the information are also needed to be carefully considered in these interventions. Involving donor and recipient families with diverse religious backgrounds and media engagement with higher visibility from the varied religious groups and even religious leaders will aid in addressing an informed decision-making, rather than the decision made in lack of information (NHSBT, 2019). Therefore, collaborative work between religious leaders, organ donation organisations, donor and recipient families, and the public in India has the potential to create an impact as demonstrated in other countries among the public (Aghaee et al., 2015; Ali et al., 2022; Randhawa & Neuberger, 2016).

## Supplementary Information

Below is the link to the electronic supplementary material.

Supplementary file1 (PDF 641 KB)
